# depressive disorder and herpes zoster oticus: a case report

**DOI:** 10.1192/j.eurpsy.2023.1595

**Published:** 2023-07-19

**Authors:** H. Chebli, A. FARH, S. belbachir, A. ouanass

**Affiliations:** University Psychiatric Hospital Arrazi in Sale, SALE, Morocco

## Abstract

**Introduction:**

The herpes zoster oticus results from the reactivation of the varicella zoster virus, a DNA virus of the Herpesviridae family with strictly human-to-human contamination, affecting the geniculate ganglion of the facial nerve. The manifestations of shingles and post-herpes signs are associated with psychiatric manifestations such as anxiety, insomnia and depressive disorder. Shingles and depressive disorder share common features, such as decreased cellular immunity and a high prevalence in the elderly

**Objectives:**

Is there a correlation between the intensity of depression and the comorbidity of herpes zoster and depression? Is there an explanation for this association? Can adequate therapy of the infection prevent the occurrence of the depressive disorder? Does the existence of this comorbidity affect the response to antidepressants?

**Methods:**

case report and litterature

**Results:**

case report

**Conclusions:**

We will try to answer these questions in this work while illustrating by the case of a patient having been touched by this comorbidity and while being based on what was published in literature.

**Disclosure of Interest:**

None Declared

